# Lighting Up the Fire in the Microenvironment of Cold Tumors: A Major Challenge to Improve Cancer Immunotherapy

**DOI:** 10.3390/cells12131787

**Published:** 2023-07-05

**Authors:** Alice Benoit, Guillaume Vogin, Caroline Duhem, Guy Berchem, Bassam Janji

**Affiliations:** 1 Tumor Immunotherapy and Microenvironment (TIME) Group, Department of Cancer Research, Luxembourg Institute of Health (LIH), L-1210 Luxembourg, Luxembourg; alice.benoit@lih.lu (A.B.); berchem.guy@chl.lu (G.B.); 2 Centre National de Radiothérapie François Baclesse, L-4005 Esch-sur-Alzette, Luxembourg; guillaume.vogin@baclesse.lu; 3 Ingénierie Moléculaire et Physiopathologie Articulaire (IMoPA), Université de Lorraine—UMR 7365, 54505 Vandoeuvre-lès-Nancy, France; 4 Department of Hemato-Oncology, Centre Hospitalier du Luxembourg, L-1210 Luxembourg, Luxembourg; duhem.caroline@chl.lu; 5 Faculty of Science, Technology and Medicine, University of Luxembourg, L-4367 Belvaux, Luxembourg

**Keywords:** immunotherapy, immune checkpoint inhibitors, tumor microenvironment, metabolic reprogramming, hypoxia

## Abstract

Immunotherapy includes immune checkpoint inhibitors (ICI) such as antibodies targeting cytotoxic T-lymphocyte-associated protein 4 (CTLA-4) or the programmed cell death protein/programmed death ligand 1 (PD-1/PD-L1) axis. Experimental and clinical evidence show that immunotherapy based on immune checkpoint inhibitors (ICI) provides long-term survival benefits to cancer patients in whom other conventional therapies have failed. However, only a minority of patients show high clinical benefits via the use of ICI alone. One of the major factors limiting the clinical benefits to ICI can be attributed to the lack of immune cell infiltration within the tumor microenvironment. Such tumors are classified as “cold/warm” or an immune “desert”; those displaying significant infiltration are considered “hot” or inflamed. This review will provide a brief summary of different tumor properties contributing to the establishment of cold tumors and describe major strategies that could reprogram non-inflamed cold tumors into inflamed hot tumors. More particularly, we will describe how targeting hypoxia can induce metabolic reprogramming that results in improving and extending the benefit of ICI.

## 1. Introduction

Immunotherapy is a treatment that aims to reactivate the immune system of cancer patients to fight tumor cells. Such therapy includes adoptive T cell therapy, anti-cancer vaccination, oncolytic viruses, and general immunotherapy consisting of the administration of interferon gamma or interleukin-2 as well as immune checkpoint inhibitors (ICI).

Briefly, immune checkpoint inhibitors are monoclonal antibodies directed against immune checkpoint receptors expressed mostly on immune cells or against their ligands expressed on tumor cells. Preventing the interaction between the immune checkpoints and their ligands modulates the function of immune cells against cancer cells. Some receptors are described as co-activators and will strengthen the activation of effector cells such as a cluster of differentiation (CD) 28 and its ligand CD80. In contrast, some receptors are called co-inhibitors, which will decrease the activation of effector cells such as programmed death protein 1 (PD-1) and its programmed death ligands PD-L1/PD-L2 [1]. Cytotoxic T lymphocytes express both types of co-activator and co-inhibitory receptors, and the complex balance between the activation and inhibition signals provided by these receptors will shape the activation state of immune cells. Indeed, tumor cells are capable of manipulating immune checkpoints to their advantage by over-expressing inhibitory receptor ligands at their surface. Thus, cancer cells can inhibit the activation of the T cell effector and escape the immune system [2].

Immune checkpoint inhibitors used in cancer therapy are mostly directed toward two major targets: (1) cytotoxic T lymphocyte antigen 4 (CTLA-4) and (2) PD-1/PD-L1. The first generation of immune checkpoints inhibitor (Ipilimumab) was directed against CTLA-4. CTLA-4 is expressed on CD4+, CD8+ T lymphocytes, regulatory T cells, and T helper cells. When the T cell receptor (TCR) of T lymphocytes recognizes a specific antigen, CTLA-4 will be produced according to the strength of the TCR signal. CTLA-4 has the same ligands as the co-activator of the CD28 receptor: CD80 and CD86. Since CTLA-4 presents a higher affinity for CD80 and CD86 ligands than CD28, the expression of CTLA-4 will inhibit lymphocyte activation [3].

The second generation of immune checkpoint inhibitors was directed against PD-1 or its ligand PD-L1. Anti-PD-1 antibodies, such as Nivolumab and Pembrolizumab, are used in the clinic for the treatment of melanoma, lung, kidney, bladder, head, and neck cancers as well as Hodgkin’s lymphoma. Anti-PD-L1 agents such as Atezolizumab, Durvalumad, and Avelumab are indicated for the treatment of bladder and lung cancers as well as Merkel carcinoma. PD-1 and CTLA-4 are also expressed by CD8+, CD4+ T cells, and T regulatory (Treg) cells in the same manner as CTLA-4. Although PD-1 has two ligands (PD-L1 and PD-L2), the PD-1/PD-L1 axis seems to be the main mechanism used by cancer cells to escape the immune system [3].

ICIs have emerged as a promising new treatment yielding significant survival benefits for patients in whom other conventional therapies have failed. However, the response to immunotherapy is relatively heterogeneous according to the cancer type, and the benefits remain low for most patients [4]. Certain types of immunotherapies display severe autoimmune side effects such as the CTLA-4-specific antibody ipilimumab despite the considerable efforts made to uncouple its toxicity and efficacy and potentiate its role as a depleting antibody of regulatory T cells [5]. The limited clinical benefits for a relatively small percentage of patients rely on several factors including the absence or limited infiltration of immune cells within the microenvironment [6].

The immunoscore concept refers to the type, density, and location of immune cells in the tumor microenvironment. The immunoscore has been used to classify tumors as hot, altered-excluded, altered-immunosuppressed, and cold. This classification is based on an immunoscore from zero to four depending on the quantification of CD3 and CD8 T lymphocytes. Cold tumors have an immunoscore of zero and present no infiltration of immune cells within the tumor and the invasive margin, while hot tumors have an immunoscore of four and present high infiltration of immune cells in both areas. Altered or warm tumors have low infiltration of immune cells, either in the tumor margin only (altered-excluded tumors) or in both areas (altered-immunosuppressed tumors) [7,8].

Cold, altered, and warm tumors present low or no clinical benefits from the use of immune checkpoint inhibitors. Therefore, reprograming these tumors into hot, inflamed, and immune-infiltrated tumors is now a well-defined strategy to improve the patient’s response to anti-CTLA-4 and anti-PD-1/PD-L1 immunotherapy. This review will summarize some tumor characteristics associated with the lack of immune cell infiltration within the microenvironment of cold tumors. We will also focus on emerging strategies for reprogramming non-inflamed cold tumors into inflamed hot tumors, with a focus on metabolic reprogramming by targeting hypoxia.

## 2. Factors Involved in the Establishment of Immune Desert Tumors

In this section, we will discuss the main factors contributing to the establishment of cold desert immune tumors.

### 2.1. Defects in the Cancer-Immunity Cycle

**Figure 1:**
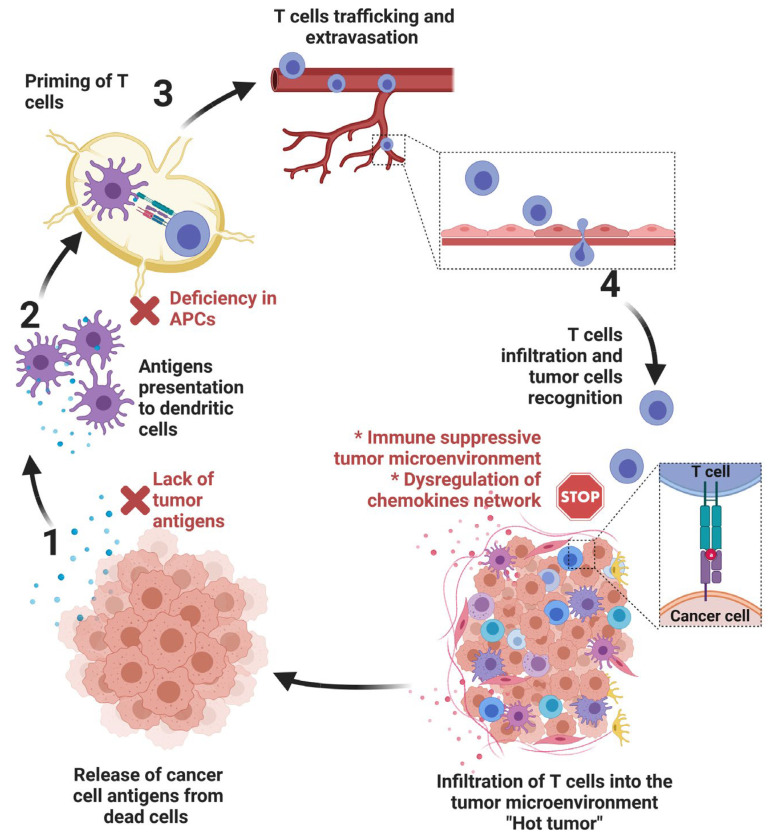
The different steps of the cancer-immunity cycle. Briefly, the first step of the cycle consists of releasing antigens from tumor cells (1). These antigens are captured by dendritic cells on MHC-I and MHC-II molecules (2) and presented to T cells (3). This step results in T cell priming and activation in lymph nodes. Activated T cells migrate through the blood vessels to infiltrate the tumor microenvironment (4). Defects in several steps in the cancer-immunity cycle results in the establishment of cold tumors (reported in red).

The cancer immune cycle contains multiple steps. The first step is the production of tumor antigens by cancer and normal cells. These antigens are then recognized and presented to the effector T cells by antigen-presenting cells (APCs). T cells are then activated and co-stimulated, leading to an anti-tumoral response toward cancer cells. The absence of effector immune cells infiltration within the tumor microenvironment could relate to (i) a lack or low expression of tumor antigens, (ii) a deficiency in the recruitment of APCs that result in a decrease in T cell activation and co-stimulation, (iii) an immunosuppressive tumor microenvironment, and (iv) the dysregulation of the cytokine/chemokine network [9]. In this section, we will briefly describe how each step of the cancer-immunity cycle contributes to the establishment of immune desert or cold tumors (Figure 1).

#### 2.1.1. Low Expression of Tumor Antigens

Recognition of tumor antigens constitutes a key step of the anti-tumor immune cycle. Tumor antigens can be categorized into two classes: (1) tumor-specific antigens (TSAs) produced by cancer cells only and not healthy cells, and (2) tumor-associated antigens (TAAs) that can be produced by both cancer and normal healthy cells but are present on elevated levels on tumor cells. These antigens are generated from a wild range of genes that have undergone diverse mutations to ultimately express abnormal proteins. Basically, antigens are necessary to trigger immune responses and unleash adaptive immune responses after repeated presentation of these antigens. Following their recruitment, APCs will be in charge to present antigen fragments to T cells to activate them to kill cancer cells and eliminate tumors. However, the immune system cannot recognize the majority of tumor antigens because they are considered to be self-proteins [10].

We can distinguish different types of TAAs. These include overexpressed proteins or lineage-specific markers of differentiation expressed by tumors as well as cancer germline antigens (CGAs) expressed by tumors, trophoblasts, testis, and fetal ovaries. For TSAs, we can differentiate oncoviral antigens that are expressed by virus-associated tumors such as cervical cancer caused by HPV, and neoantigens that are produced by tumor cells due to genomic mutations [10].

As previously reported, TAAs are self-antigens that are not mutated and expressed in healthy tissues; thus, there is a lack of immunogenicity and tolerance by immune cells. In addition, targeting these TAAs with high-affinity TCRs could cause toxicity and destruction of normal tissues expressing these antigens because of a so-called “on-target toxicity.” This could also cause unintentional cross-reactivity expressing normal proteins with a similar structure called “off-target toxicity” [11]. A well-known example of tumor-associated antigens from the self is the human epidermal growth factor receptor 2 (HER2)—a transmembrane receptor involved in the regulation of cell proliferation. In HER2-positive breast cancer and other types of cancers, HER2 is overexpressed and promotes cancer cell proliferation and survival. Tumor mutational burden (TMB) is defined as the total number of mutations per analyzed tumor genomic region. TMB has been proposed as a biomarker of the response to anti-PD-1 therapy [12]. The low number of neoantigens or tumor mutational burden is frequently associated with a weak response to immunotherapy.

#### 2.1.2. Deficiency in APC Recruitment and Maturation/Activation

Following the recognition of tumor antigens, the second step of the immune response is the presentation of these antigens by APCs to T cells. Several subtypes of APCs have been characterized including conventional dendritic cells (cDC) type-1 (cDC1s) and type-2 (cDC2s), plasmacytoid DC (pDCs), monocyte-derived DCs (MoDCs) also known as inflammatory DC (iDCs), macrophages, and Langerhans cells. Although each subtype of APC is responsible for different functions in the immune response, the type-1 DCs (cDC1s) are the most important for the anti-tumor immune response because they are responsible for inducing anti-tumor CD8 T cell responses after the presentation of tumor antigens via the class I major histocompatibility complex (MHC-I) [13]. It is now well established that the cross-presentation of antigens by cDC1s promotes a key CTL response in the tumor microenvironment, although other subsets of dendritic cells such as monocyte-derived dendritic cells (moDCs) can also participate in the cross-presentation of antigen and CTL priming [14]. In addition, cDC1s are important in the response to PD-L1 therapy [15], and patient survival has been positively correlated with the abundance of tumor-infiltrating cDC1, making cDC1 a key factor in the anti-tumor response [15,16,17].

DCs need to be activated to induce an effective T cell-mediated anti-tumor response. This activation is characterized by the expression of pattern recognition receptors (PRRs) and binding of pathogen-associated molecular patterns (PAMPs) or danger-associated molecular patterns (DAMPs) [18]. Low production or absence of PAMPs or DAMPs could be related to the lack of dendritic cell maturation and subsequently the absence of naïve T cell priming/activation. Moreover, activation of APCs is driven by stimulation of their CD40 receptors. Such stimulation regulates the expression of co-activator receptors CD80 and CD86, which are present on T cells [19]. Subsequently, a defect in APC stimulation leads to a reduction in anti-tumor responses driven by CD8+ T cells.

#### 2.1.3. Immunosuppressive Tumor Microenvironment

Suppressive immune cells such as tumor-associated macrophages (TAMs), cancer-associated fibroblasts (CAFs), Tregs, and MDSCs are major components involved in establishing an immunosuppressive tumor microenvironment. TAMs are recruited to the tumor microenvironment and undergo polarization to generate macrophages displaying anti-tumoral phenotype (M1) or macrophages exhibiting a pro-tumoral phenotype (M2) [20]. The pro-tumoral M2 macrophages promote angiogenesis, metastasis, as well as key signaling pathways involved in treatment resistance [21]. In contrast to normal fibroblasts which are activated following the wound healing process, inflammation, or fibrosis, CAFs are constitutively-activated stromal cells producing ECM, growth factors, and cytokines to promote angiogenesis and recruit immune suppressive cells [22]. Tregs are among the most abundant suppressive cells in the tumor microenvironment. They have a key role in maintaining immune homeostasis, and their abundance is associated with poor prognosis for cancer patients [23]. Tregs are also associated with cancer progression, invasion, and metastasis; they are valuable targets in the treatment of cancer [24]. Finally, MDSCs are major regulators of cancer progression by secreting cytokines such as TGF-β, VEGF, and MMP9 to promote angiogenesis and metastasis. MDSCs also interact with NK, T cells, and macrophages to exert immunosuppressive effects and inhibit their activation. MDSCs promote the proliferation and activation of Tregs [25].

In addition to the presence of immunosuppressive cells, several pathways have been identified as key components involved in T cell exclusion. Indeed, a loss of phosphatase and tensin homolog (PTEN) has been correlated with the expression of immunosuppressive cytokines and decreased T cell infiltration to the tumor microenvironment in addition to an increase in tumor resistance to immunotherapy [26]. Another pathway involved in T cell exclusion is the WNT/β-catenin pathway. In melanoma, tumor-intrinsic active β-catenin signaling has been associated with the absence of type-1 DCs and effector CD8+ T cells in addition to resistance to anti-PD-L1 and anti-CTLA-4 therapy [27].

Together with the suppressive immune cells described above, hypoxic stress in the tumor microenvironment plays a major role in immune resistance and contributes to the impairment of immune cell-mediated tumor killing [28,29,30,31,32]. Emerging new data demonstrates that hypoxia is involved in the alteration of tumor metabolism and metabolites, pH regulation, and overexpression of several immune checkpoints including macrophage immune checkpoints CD47, PD-L1, and HLA-G [29,30,32,33]. Manipulating hypoxia is thus a valuable target for enhancing anti-tumor immune responses. Targeting hypoxia or hypoxia-downstream pathways has been reported to enhance T cell- and NK-cell-mediated tumor cell killing (reviewed in [34,35,36]).

#### 2.1.4. Dysregulation of the Chemokines and Cytokines Network

The chemokine network is an important contributor to immune cell trafficking and immune infiltration. In the context of cancer, inflammatory pathways are dysregulated, resulting in enhanced chemokine release. This dysregulation has an impact on cancer progression by affecting the infiltration of immune cells into the tumor microenvironment. Indeed, it has been reported that the chemokine ligands CXCL9, 10, 11, and 16 as well as CX3CL1 are responsible for recruiting T cells and natural killer (NK) cells into the tumor. CCL19 and 21 can promote the recruitment of DCs into T cell priming sites, thus leading to T cell activation [37]. Higher levels of CXCL16 have been associated with increased tumor-infiltrating lymphocytes (TILs) and better prognosis in colorectal cancer [38]. In melanoma, the chemokines CCL2, 3, 4, and 5 as well as CXCL9 and 10 were reported to induce effective T cell migration into the tumor microenvironment [39]. Strategies are being developed to increase the secretion of pro-inflammatory chemokines and improve the infiltration of immune cells in the tumor such as targeting HIF-1α, which resulted in an increase in the pro-inflammatory chemokine CCL5 and an increase in the infiltration of CD4+ and CD8+ T cells as well as NK cells [40].

Several cytokines influence cancer progression and treatment response. For instance, transforming growth factor-β (TGF-β) contributes to the lack of T cell infiltration and low response to anti-PD-1/PD-L1 immunotherapy in metastatic urothelial cancer [41] and colon cancer metastasis [42]. The TGF-β blockade in combination with anti-PD-L1 treatment contributes to an increase in T cell infiltration and improvement in tumor regression in mouse models [41]. Besides TGF-β, IFNγ, IL-2, and IL-9 also contribute to the efficacy of anti-PD-1 therapy [43].

## 3. Strategies to Turn Non-Inflamed Cold Tumors into Inflamed Hot Tumors

**Figure 2:**
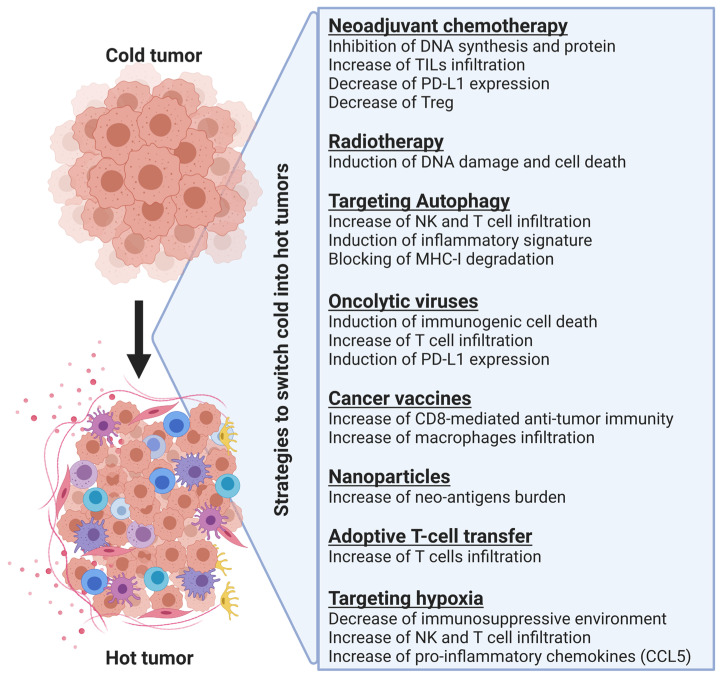
Summary of the different strategies involved in switching cold into hot tumors and their mode of action. Combining these strategies with immunotherapy improves the therapeutic benefit in cancer patients.

As described previously, switching non-inflamed cold tumors into inflamed hot tumors and thus increasing T cell infiltration into the tumor microenvironment is a promising strategy for improving cancer immunotherapy. Below, we will summarize strategies already described for modulating the immune phenotype of different tumors and discuss briefly how these strategies improve the therapeutic benefits of anti-PD-1/PD-L1 and anti-CTLA-4-based therapy (Figure 2).

### 3.1. Neoadjuvant Chemotherapy

Chemotherapy is a conventional oncology treatment for inducing tumor cell death and preventing tumor growth by inhibiting multiple processes such as DNA synthesis or protein function. Experimental and clinical evidence show that chemotherapy could have an impact on the tumor immune landscape, thus resulting in an improvement in immunotherapy. In this context, it has been reported that neoadjuvant chemotherapy could increase the number of tumor-infiltrating lymphocytes (TILs) within the tumor and decrease PD-L1 expression in residual breast cancer tissues [44]. Another study showed a correlation between adjuvant chemotherapy and decreased regulatory Treg infiltration in breast cancer; there was also an increase in cytotoxic T cell infiltration [45]. Neoadjuvant chemotherapy had the same effects in non-small cell lung carcinoma (NSCLC) in which the number of tumor-associated immune cells was higher in tumors from patients receiving neoadjuvant chemotherapy compared to tumors from untreated patients [46]. Altogether, these data suggest that chemotherapy can induce an anti-tumor immune response and that neoadjuvant chemotherapy could enhance the benefits of anti-PD-1/PD-L1 and CTLA-4 immunotherapy.

The combination of chemoradiotherapy and immunotherapy is currently under investigation in a phase-II clinical trial with patients with locally advanced cervical cancer [47]. For patients with lung squamous cell carcinoma, the combination of chemotherapy with radiotherapy resulted in a longer overall survival and progression-free survival; however, this strategy increased the risk of gastrointestinal abnormalities, hematoxicity, and liver dysfunction [47]. For patients with non-metastatic and metastatic esophageal cancer, neoadjuvant chemoradiotherapy and immunotherapy have no effect on the survival benefits [48]. In addition, chemotherapy is not a specific treatment targeting cancer cells only—it results in cardiac, dermatologic, gastrointestinal, hepatic, nephrotic, and pulmonary toxicities. It also has cytotoxic effects on immune cells and can damage the bone marrow, hence decreasing the production of immune cells and impairing the anti-tumor immune response [49]. Considering the high toxicity and the few effects observed in clinical settings, this suggests that neoadjuvant chemotherapy might not be the best candidate to improve immune infiltration and immunotherapy.

### 3.2. Radiotherapy

Radiotherapy is a conventional therapy for many cancers. The primary aim of radiotherapy is to induce DNA damage in cancer cells, which are dividing more rapidly than healthy cells. The ultimate goal is to induce tumor cell death through mitotic catastrophe or apoptosis. Radiotherapy also does not specifically target cancer cells, but unlike chemotherapy, radiotherapy focuses specifically where the cancer is located and causes fewer side effects in general. The course of treatment will depend on the type and location of the pathology as well as the overall health status. Several studies showed that radiotherapy can induce an immune response [50] even for primary and metastatic brain cancers that are located in an area lacking appropriate immune surveillance [51]. In addition, radiation induces the expression of mutant proteins and potentially mutant neoantigens, which can generate a CD8+ T cell response [52]. Furthermore, radiotherapy induces ferroptosis, necroptosis, and pyroptosis, which can stimulate a post-radiation immune response [53].

The concept of enhancing the benefits of immunotherapy treatments via adjuvant radiotherapy has been shown in several studies. Briefly, in a breast carcinoma mouse model, dual treatment with fractioned radiotherapy and an anti-CTLA-4 antibody enhanced the abscopal effect [54], which refers to tumor regression outside the field of radiation due to an indirect systemic anti-tumoral effect induced by radiotherapy [55]. In this study, the effect was associated with a higher frequency of CD8+ T cells showing tumor-specific IFN-γ production. This was translated in clinical settings with a case report of a patient with small-cell lung carcinoma who was treated with a combination of radiation therapy and nivolumab [56]. In murine osteosarcoma, radiotherapy could enhance antitumor efficacy of anti-PD-L1 treatment with a significant decrease in tumor growth and an improved overall survival via an increase in PD-1-positive and granzyme B-positive CD8+ T cells [57].

In a Lewis lung carcinoma mouse model, the use of an anti-CTLA-4 antibody led to an enhancement of the anti-tumor activity of irradiation by delaying tumor growth and prolonging mice survival [58]. Similar results were observed in poorly immunogenic metastatic mouse mammary carcinoma treated with a combination of radiotherapy and CTLA-4 blockade. Here, the combination treatment was also effective in inhibiting metastasis formation via CD8+ T cells [59]. In advanced melanoma, combining radiation therapy with ipilimumab resulted in improved patient survival and complete response rates [60]. Other clinical data concerning melanoma patients have provided evidence that the combination of radiotherapy with a dual-checkpoint blockade, including anti-CTLA-4 and anti-PD-L1 antibodies, could promote an immune response through distinct mechanisms. Anti-CTLA-4 antibodies inhibit Tregs and increase the CD8+ T cells-to-Tregs ratio, while the PD-L1 blockade reverses T cell exhaustion; radiation enhances the diversity of TCRs [61]. However, when radiotherapy was assessed in combination with anti-PD-L1 and anti-CTLA-4 antibodies for non-small-cell lung carcinoma patients in a phase-II clinical trial, there was no difference in the response rate or the progression-free survival [62]. The challenge for radiotherapy prior to immunotherapy remains in determining the optimal sequence, volume, and radiation dosage.

### 3.3. Targeting Autophagy

Autophagy is a highly regulated cellular process that is involved in the recycling and degradation of cytoplasmic contents. In cancer, experimental and clinical evidence shows that autophagy acts as a cytoprotective mechanism. Emerging preclinical data show that targeting the autophagy-related gene Beclin-1 inhibits tumor growth and induces the infiltration of functional NK cells into the microenvironment of melanoma tumors in a CCL5-dependent manner [63,64].

More recently, genetic and pharmacological targeting of the vacuolar protein sorting 34 (Vps34; involved in the initiation of autophagy or in the process of endocytosis) has been shown to impact the immune landscape of melanoma and colorectal cancer. Indeed, Vps34 inhibition induces the infiltration of NK, CD8+, and CD4+ T effector cells to the tumor microenvironment. Furthermore, a combined treatment with Vps34 inhibitors and anti-PD-1/PD-L1 antibodies improves the therapeutic benefits in tumor-bearing mice compared to immunotherapy alone. These data provide evidence that Vps34 inhibition improves colorectal cancer and melanoma sensitivity to immune checkpoint blockade [63,64,65,66,67]. Recent data highlight that autophagy is involved in selective degradation of the MHC-I molecules in the lysosome compartment through the cargo receptor NBR1 in pancreatic ductal adenocarcinoma. The inhibition of autophagy caused a restoration of MHC-1 expression and an improvement in antigen presentation that resulted in enhanced T cell responses and a subsequent decrease in tumor growth. Genetic and pharmacological inhibition of autophagy was shown to improve anti-PD1 and anti-CTLA4 antibody-based immunotherapy [68].

To date, chloroquine and its derivatives are the only FDA-approved drugs for targeting autophagy. It has been shown that chloroquine had a synergistic effect when combined with anti-PD-1 and anti-CTLA-4 antibodies in mouse models of pancreatic cancer [69]. Hydroxycholoroquine is currently investigated in several clinical trials in combination with different immunotherapy molecules for metastatic melanoma, gastrointestinal cancer, and pancreatic cancer. Therefore, targeting autophagy at multiple steps is now an emerging and promising strategy for converting cold tumors into hot inflamed tumors.

### 3.4. Oncolytic Viruses

Oncolytic viruses are agents that preferentially kill cancer cells via oncolysis. The first oncolytic virus approved for cancer treatment in the United States and Europe was a modified herpes simplex virus called talimogene laherparepvec or T-VEC. It was approved in 2015 by the Food and Drug Administration (FDA) for the treatment of advanced melanoma. While oncolytic viruses were mainly used for their capability of killing cancer cells, several studies have shown that they are also capable of promoting anti-tumor immunity. For high-grade gliomas, an intravenous infusion of the oncolytic human Orthoreovirus in patients could increase the cytotoxic T cell infiltration within the tumors, IFN gene expression, and expression of PD-1 and PD-L1, thus suggesting that the combination of this reovirus with PD-1/PD-L1 blockade could lead to significant clinical benefits [70]. For triple-negative breast cancer, the administration of the oncolytic virus Maraba offered long-term benefits prior to surgery and sensitized tumors to immune checkpoint blockade in preclinical animal models. This oncolytic virus was also capable of promoting intra-tumoral infiltration of cytotoxic immune cells and inducing the expression of PD-L1 [71]. More recently, the engineering of an oncolytic virus expressing PD-1 inhibitors led to improved T cell activity in humanized PD-1 mouse models with an increased anti-tumor effect. This effect was related to an increased infiltration of CD8+ T cells, the establishment of memory CD8+ T cells, and a reduction in CD8+ T cell exhaustion. These results were improved when associating anti-CTLA-4 or anti-TIM-3 without any evidence of neurotoxicity in non-human primates [72].

The use of an oncolytic adenovirus loaded with interleukin 7 has already shown promising results in clinical settings and increases tumor-infiltrating lymphocytes. There is decreased tumor growth by up-regulating pro-inflammatory cytokines and inducing the activation and migration of CD4+ and CD8+ T cells [73]. Moreover, the use of ferroptosis (iron-dependent programmed cell death) inducers in combination with an oncolytic vaccinia virus induced a stronger therapeutic effect than each treatment alone. There was an increase in the number and activity of tumor-infiltrating lymphocytes in hepatoma, colon, and ovarian cancer cells [72]. These data collectively suggest that the use of oncolytic viruses could improve the immunotherapy response especially in combination with PD-1/PD-L1 antibodies. The use of oncolytic viruses is also a promising strategy for the most lethal malignancies that are not responding to classical treatment strategies such as pancreatic cancer [74], gastrointestinal malignant tumors [75], and glioblastoma [76]. However, efforts are still needed to improve the drug delivery, bioavailability, and the cost of production of oncolytic viruses [77].

### 3.5. Vaccines

Vaccines often use a benign form of the pathogenic agent injected into humans to stimulate an immune response. It has been reported that an intratumor injection of the seasonal flu shot in melanoma mice model can generate antitumor immunity mediated by CD8+ effector T cells. In addition, the neoadjuvant flu shot injection could improve tumor responses to anti-PD-L1 immune checkpoint blockade compared to anti-PD-1 alone [78]. Another study used a biomaterials-based strategy for converting tumor-derived antigenic microparticles into a cancer vaccine. Interestingly, this vaccine was capable of inducing a strong immune response with high infiltration of tumor-associated macrophages and CD8+ T cells within the tumors. The combination of this microparticle vaccine with PD-L1 blockade in a melanoma mouse model inhibited tumor progression and improved mouse survival [79].

The mRNA vaccines against SARS-CoV-2 have been repurposed for cancer treatment. Lipid-based nanoparticles delivering mRNA could stimulate a T cell response without conferring changes in the spleen and liver of non-human primates [80]. The mRNAs are transcribed in vitro (IVT) to structurally resemble natural mRNA. The IVT mRNAs are delivered by lipid nanoparticles, thus allowing recipient cells to produce many copies of the encoded proteins that can stimulate potent immune responses [81]. The development of the mRNA vaccine technology has gained significant interest in the field of cancer treatment. In fact, several clinical trials are ongoing to evaluate the potential of mRNA vaccines for the treatment of a wide range of cancer types including breast cancer [82,83].

More recently, an elastic nanovaccine called SMONV (soft mesoporous organosilica-based nanovaccine) has been designed. The elasticity-dependent effect resulted in a greater internalization by dendritic cells and the stimulation of cellular and humoral immunity with a suppression of tumor growth in tumor-bearing mice when injected subcutaneously. The combination of this nanovaccine with anti-PD-L1-blocking antibodies resulted in an enhanced therapeutic effect of anti-PD-L1 [84]. Interestingly, the development of an in situ tumor vaccine expressing anti-CD47 antibodies resulted in tumor growth suppression, stronger long-term survival, and increased tumor-infiltrating lymphocytes in melanoma, lymphoma, and breast cancer mouse models [85].

To date, the only FDA-approved therapeutic anti-cancer vaccine is Sipuleucel-T, which is indicated for metastatic castration-resistant prostate cancer. This vaccine has been reported to cause the induction of CD8+ T cell infiltration in a CXCL10 manner and up-regulation of immune inhibitory checkpoints such as CTLA-4 [86], thus showing that anti-cancer vaccines are interesting leads and require further investigation [82].

### 3.6. The Use of Nanoparticles for Enhancing the Delivery of Therapies to the Tumor Site

Nanoparticles are small entities measuring less than 200 nm and consisting of liposomes, dendrimers, and nanospheres. All of these nanoparticles are described as selective carriers for anticancer agents to the tumor site. These nanoparticles are also capable of preferentially targeting the tumor microenvironment thanks to the enhanced permeation and retention (EPR) effect. Nanoparticle-based therapy has been proposed as a strategy to increase the neo-antigen burden, modulate the tumor microenvironment, and trigger the antitumor immune response with the ultimate aim of improving immunotherapy [87]. Nanomaterials can stimulate the innate and the adaptive immune system in a long-term manner by regulating the tumor immunosuppressive microenvironment [88,89,90]. Efforts have recently been made to assess the efficacy of nanoparticles to carry anti-cancer drugs in order to modulate the immune system and enhance the response to immunotherapy. This can be achieved by designing an injectable hydrogel that triggers the immune response and mitochondrial biogenesis of T cells, while enhancing MHC I expression and lowering T cell exhaustion [91]. Hydrogels were already assessed with celecoxib in combination with an anti-PD-1 antibody in B16-F10 melanoma and 4T1 metastatic breast cancer. This strategy successfully enhanced the infiltration of T cells within the tumors and reduced the presence of Treg cells and MDSCs. They could also observe an increase in CXCL9 and 10 with a decrease in IL-1, IL6, and COX2 [92]. Enhanced T cell infiltration and antigen presentation in B16-F10 melanoma was also observed when using a pH-sensitive liposome containing doxorubicin and deferasirox [93].

The emergence of immunoliposomes facilitate the conjugation of monoclonal antibodies or derivatives [94] with interesting results. A significant tumor regression of the melanoma murine model was observed by using targeted immunoliposomes containing doxorubicin and antibodies targeting PD-L1 [95]. Another system of nanoparticles can inhibit the indoleamine 2,3-dioxygenase 1 (IDO1) pathway and induce immunogenic cell death to enhance DC maturation, increase the number of CTLs, and decrease the number of Tregs in tumor tissues [96].

Nanoscience is also very promising for the treatment of glioblastoma (GBM), which is a very aggressive type of cancer with limited treatment options [97,98]. Indeed, it has been shown that cationic lipid nanoparticles can cross the blood–brain barrier, which is the main issue for drug delivery in glioblastoma. Here, they delivered siRNA against CD47 and PD-L1 in mice models of GBM [97,98,99]. Nanoparticles have also been used to target the CXCL12/CXCR4 signaling in mouse and human GBM in vitro and vivo, resulting in a decrease in GBM proliferation associated with a decreased infiltration of MDSCs and an increase in immunogenic cell death [100]. The modulation of the immunosuppressive microenvironment with the inhibition of IDO1 and JQ1, a drug decreasing the expression of PD-L1 by tumor cells, improved the immunotherapy treatment of GBM in mice [101]. The use of nanoparticles is also promising for other types of cold tumors such as colorectal cancer [102] and bladder cancer [103], where the delivery of specific drugs by nanoparticles was associated with a successful reprogramming of the tumor microenvironment with enhanced therapeutic efficacy. Together, these data show nanoparticles to be interesting and valuable tools when used as a cargo system to deliver anti-tumor drugs to the tumor site; they improve immune checkpoint blockade therapy.

### 3.7. Combining Adoptive T Cell Transfer with Other Therapies

Adoptive T cell transfer (ACT) is a type of immunotherapy where immune cells are extracted from the patient’s blood or tumor, expanded in vitro, and transfused back into the patient. Three types of ACT have been developed: tumor-infiltrating lymphocytes (TILs), T cell receptor (TCR)-engineered T cells, and CAR-T cells [104].

(i) TILs

This type of ACT is based on the isolation of TILs from patient tumor biopsy followed by in vitro stimulation with Interleukin 2 (IL-2). After in vitro expansion, the strategy consists of testing the reactivity of lymphocytes against tumors and amplifying those that are highly reactive before reinjecting them into the patient to selectively kill the remaining tumor.

This strategy has been shown to induce complete and durable regressions in metastatic melanoma patients [105]. However, it has limitations due to the downregulation of MHC molecules and the lack of infiltration of lymphocytes. Therefore, TILs only have benefits in a few cancer types—they have limited benefits (20% of reported objective response rate for metastatic melanoma) [106]. To overcome these limitations, different strategies have been tested for melanoma such as the combination of TILs with cytokines (IFN-α, IFN-β, IL-2, IL-7, IL-12, IL-15, IL-21, and TNF-α) or with vaccines (fowlpox-based vaccine, GM-CSF—producing tumor-based vaccine). These combinations showed an improved objective response in general, but also elevated toxicity with high doses of cytokines [107]. In addition to melanoma, the efficacy of TILs and anti-PD-1 combination was tested on metastatic cervical cancer without PD-L1 expression. The results show that the combination therapy of TILs and anti-PD1 significantly improves the prognosis of metastatic cervical cancer [108].

(ii) TCR-T cells

The TCR-T cells approach relies on redirecting T cell specificity. This can be achieved by expressing TCR α and β chains that can dimerize with CD3 components to form an operative TCR. Such a strategy deflects T cell specificity to recognize specific antigens in the context of HLA presentation in any subcellular compartment [109]. TCR-T cell therapy showed promising results and even significantly higher benefits compared to TILs for cutaneous melanoma [110]. They had good potential for colorectal cancer and leukemia treatment [111,112]. However, TCR-cells therapy implies several challenges such as the importance of the target antigen selection, immune escape, safety and toxicity, cytokine storms, and T cell exhaustion. Considerable efforts have been made to overcome these challenges such as improving the structural affinity of TCRs and the stromal cell selection, combining TCR-T cells with CD4+ T cells, or activating independently the costimulatory receptors (reviewed in [9]). PD-1 inhibits T cell activation by targeting the signaling of the TCR and CD28 ([9]), and thus, it would be interesting to evaluate the potential of the combination of anti-PD-1 and TCR-T cells therapy.

(iii) CAR-T cells

Chimeric Antigenic Receptor–T or CAR-T cells are structurally different to TCR-T cells. They have been designed to mimic TCR signaling with a tumor antigen-binding domain, an extracellular spacer, a transmembrane domain, costimulatory domains, and a CD3ζ signaling domain. Unlike TCR-T cells, they can bind MHC-independent ligands and show an increased binding affinity [113]. The CAR-T cell approach showed significant efficacy in acute lymphoid leukemia and lymphoma [114,115]. Dual targeting with CD19 and CD22 CAR-T cells increased toxicity and improved the remission rates of acute lymphocytic leukemia and non-Hodgkin’s lymphoma patients [116]. The efficacy of CAR-T cells in solid tumors is limited, but combinatorial approaches with oncolytic viruses expressing cytokines, chemokines, anti-PD-L1, IL-2, or TNF-α have shown that enhanced CAR-T cells affect murine models [117].

Despite major progress in the ACT approaches, additional efforts need to be performed to improve the efficacy and safety of these immunotherapies. We are also missing studies about the combination of immune checkpoint inhibitors and ACT approaches, which could provide considerable promise but could be limited by toxicity.

### 3.8. Targeting Hypoxia

As previously reported, hypoxia plays a major role in immune resistance and immune-cell mediated tumor killing. Here, we will provide an overview of the hypoxia mechanism and describe the impact of hypoxic stress on tumor response to immunotherapy. We will also summarize recent strategies developed to target hypoxia and improve patients’ response to immunotherapy.

#### 3.8.1. Hypoxia: Mechanisms and Pathways

Cancer cells adapt to hypoxia through hypoxia-inducible factors (HIFs). The HIF family contains three members: HIF-1, HIF-2, and HIF-3. HIF-1 is composed of two sub-units, HIF-1α and HIF-1β; both have basic helix–loop–helix domains and can form a heterodimer and bind to the promoter of target genes. HIF-1 is the most studied and described in the literature because of its involvement in many pathways and diseases. HIF-2α can also bind to HIF-1β, and it plays a role in vascularization, pulmonary development, and erythropoiesis [118]. HIF-3α is much less known; it is different regarding its structure and seems to act as a transcription factor competing with HIF-1α and HIF-2α [119].

**Figure 3:**
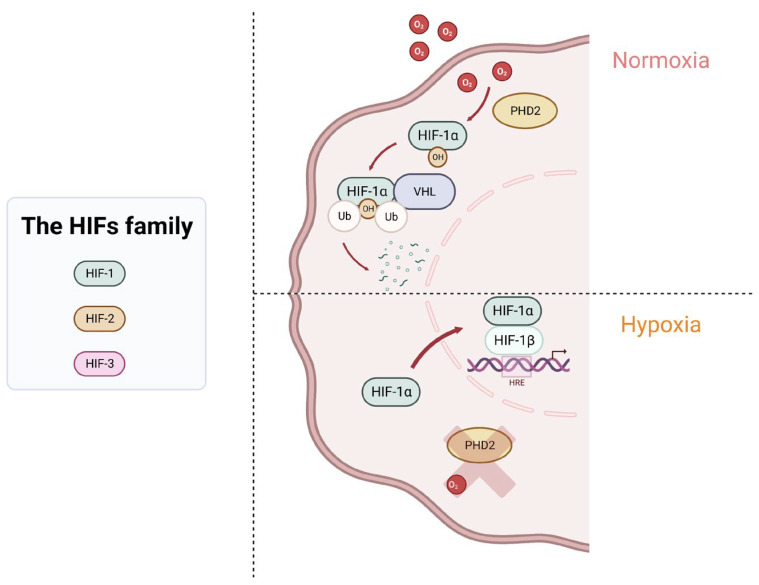
Summary of the mechanisms involved in HIF-1α regulation in normoxia and hypoxia.

In normoxic conditions, HIF-1α is hydroxylated by the prolyl hydroxylase domain protein 2 (PHD2), and the complex interacts with the Von Hippel–Lindau tumor suppressor protein (VHL). The VHL protein is part of a ubiquitin-ligase complex E3 that is involved in the hydroxylation and polyubiquitination of HIF-1α, thus leading to its degradation by the proteasome system. In hypoxic conditions, the low level of oxygen inhibits PHD2’s activity, which prevented hydroxylation and the degradation of HIF-1α. HIF-1α accumulates in the cytoplasm before being translocated into the nucleus. In the nucleus, HIF-1α forms a dimer with the subunit HIF-1β, and the heterodimer binds to the hypoxia response elements (HRE) motif in the promoter of different target genes to activate their transcription [120,121] (Figure 3).

#### 3.8.2. Hypoxia Inducible Factors and Cancer

HIFs play a central role in many physiological conditions because they are responsible for oxygen homeostasis, but they are also involved in pathological processes such as cancer. A high expression of HIF-1α and/or HIF-2α has been associated with an increased risk of mortality in more than 10 different types of cancer [118]. Target genes of HIFs include many genes involved in different steps of cancer biology such as cell immortalization, genetic instability, epithelial-to-mesenchymal transition, angiogenesis, metabolism, invasion and metastasis, and, more importantly, immune evasion [122].

Hypoxia influences tumor immune escape through four different aspects: (i) regulation of signal transduction pathways, (ii) suppression of immune effector cells, (iii) recruitment of immunosuppressive cells and upregulation of inhibitory immune checkpoints, and (iv) activation of autophagy [122].

(i) Regulation of signal transduction pathways

As previously reported, HIFs can bind to the HRE motifs found in several genes including the vascular endothelial growth factor (VEGF), which is a key mediator of angiogenesis in embryogenic development and in wound healing. In cancer, VEGF is up-regulated, allowing the formation of blood vessels to provide nutrients and oxygen to the tumor. In addition, vessels formed under these conditions are chaotic, leaky, and not well-structured. These characteristics lead to generating more hypoxia and thus more VEGF production [123]. The HIF-1α/VEGF axis in cytotoxic CD8+ T cells regulates tumor progression, and the deletion of VEGF-A in CD8+ T cells accelerated tumorigenesis while also altering vascularization [120].

About 75% of clear cell renal cell carcinomas display a loss of function of the VHL gene, which results from mutations, loss of heterozygosity, and promoter methylation [124,125]. The loss of function of VHL leads to a constitutive stabilization of HIF-1α and HIF-2α, which induces the activation of HIF-target genes and pathways involved in cancer progression.

HIFs also play a role in mitochondrial metabolism because HIF-1α activation induces the pyruvate dehydrogenase kinase 1 to deprive mitochondria from pyruvate. HIF-1α also induces BNIP3, which is responsible for triggering mitochondrial selective autophagy and CD73, which is an enzyme producing the immunosuppressive factor adenosine [126]. HIF-1 is also responsible for the up-regulation of glucose transporter proteins (GLUTs) in cancer cells to promote glycolysis [127] and the induction of epithelial to mesenchymal transition (EMT) in several types of cancer, thus promoting tumor progression and metastasis [128,129].

The tumor microenvironment displays dysregulated vasculature and glycolytic tumor cells. It contains hypoxic regions with an accumulation of metabolic products such as lactate. Lactate is the most increased metabolite in solid tumors [130]. The accumulation of lactate decreases the pH, which ultimately impairs almost all features of CD4+ and CD8+ T lymphocytes (proliferation, chemotaxis, activation, and cytotoxicity). Lactate also promotes the development of immunosuppressive Tregs and immune cell exhaustion due to the switch in metabolic activity and the lack of nutrients within the tumor microenvironment [131].

(ii) Suppression of immune effector cells

HIF-1 has a negative effect on MHC-I expression, thus limiting tumor cell recognition by T cells [132,133]. As previously reported, HIF-1α increases the expression of a wide range of immune checkpoints such as CTLA-4 on T cells; LAG3, TIM3, and PD-L1 on tumor cells; and PD-L1 and VISTA on MDSCs, thus resulting in the inhibition of T cell proliferation and T cell-mediated lysis. CD47 is also up-regulated by hypoxia on tumor cells, thus resulting in the inhibition of tumor cells’ phagocytosis. Finally, hypoxia triggers the activation of autophagy, thus impairing tumor cell susceptibility to NK and CTL-mediated killing [134].

(iii) Recruitment of immunosuppressive cells

Hypoxia contributes to the recruitment of immunosuppressive cells—notably tumor-associated macrophages (TAMs), Tregs, and myeloid-derived suppressor cells (MDSCs). In Tregs, hypoxia induces the secretion of the chemokine CCL28, which is responsible for their recruitment to the tumor microenvironment [135]. Hypoxia also promotes the suppressive function of MDSCs by inducing the expression of VISTA on myeloid cells [136]. Likewise, hypoxia promotes the tumor-supporting functions of TAMs via the up-regulation of iron exporter ferroportin (FPN) and lipocalin 2. This leads to increased iron availability and thus proliferative activation of cancer cells as well as the up-regulation of the regulated in development and DNA damage responses 1 (REDD1)—an inhibitor of MTOR complex 1. This results in a shift toward oxidative metabolism and increased glucose availability in the tumor microenvironment [137].

(iv) Induction of autophagy

Hypoxia can induce cytoprotective autophagy via the expression of BNIP/BNIP3L, leading to the dissociation of the Bcl-2/Beclin-1 complex to activate autophagy (reviewed in [138]). Interestingly, blocking hypoxia-induced autophagy restored cytotoxic T cell activity and promoted tumor regression [139].

#### 3.8.3. Targeting Hypoxia as a Strategy to Improve Patient’s Response to Immunotherapy

Hypoxia is responsible for tumor escape from immune surveillance [35,140]. It has been demonstrated that HIF1-α can bind to the HRE motif of the promoter of PD-L1 gene and induce its expression in prostate, breast, and lung cancer as well as melanoma [30]. In addition to PD-L1, hypoxia induces the V-domain immunoglobulin suppressor of T cell activation (VISTA) expression by MDSCs and promotes their suppressive function. VISTA is an immune checkpoint, and its expression on tumor cells and/or immune cells is associated with an immunosuppressive tumor microenvironment in several cancer types. Targeting of VISTA by antibody or genetic ablation under hypoxia decreased MDSC-mediated T cell suppression, thus suggesting that combination approaches targeting both VISTA and hypoxia could reverse the immunosuppressive antitumor immunity, and thus improve immunotherapy [141].

The emergence of hypoxia-activated prodrugs provides an interesting strategy to target hypoxia in solid tumors. The most studied one is TH-302, also called Evovosfamide. The activation of TH-302 relies on genes involved in mitochondrial electron transfer, DNA damage-response factors, and mitochondrial function regulators. The activation of TH-302 occurs under hypoxia based on the reduction in free radical anions, leading to the release of bromo-iso-phosphoramide mustard (Br-IPM) or its stable downstream product, isophosphoramide mustard (IPM) [142]. In prostate cancer, it has been reported that combining immune checkpoint blockades with the hypoxia-activated prodrug TH-302 significantly inhibits tumor growth and extends survival in a transgenic adenocarcinoma prostate-derived mouse model. This effect was associated with a decrease in MDSCs in the tumor microenvironment, and a decrease in intra-tumoral hypoxia [143]. The drug has shown relevant potential when used in combination with cytotoxic drugs, targeted therapy, or radiotherapy [144]. TH-302 is currently under investigation in several clinical trials for different types of cancer [142].

In addition to hypoxia-activated prodrugs, nanoparticles can also target hypoxic tumor microenvironments. In the blood circulation, the hypoxia-responsive nanoparticles are inactive and become active once they reach the hypoxic zones of the tumor. They can be used to boost the efficiency of different treatments such as chemotherapy and radiotherapy [145]. For instance, in hepatocellular carcinoma, the use of the FDA-approved nanoparticle with photothermal abilities could alleviate tumor hypoxia, trigger an antitumor immune response, inhibit tumor metastasis, and induce long-term immunological memory when combined with anti-PD-L1 immunotherapy [146]. Nanoparticles are also considered to target hypoxia in glioblastoma [56,99,100,146].

Considering the involvement of hypoxia in the immunosuppressive microenvironment of tumors, we provided evidence that deleting the transcriptional activity of HIF-1α decreased the tumor growth of a melanoma mouse model and increased the infiltration of CD45+, NK, CD4+, and CD8+ cells. Such infiltration was related to an increase in the release of CCL5 in HIF-1α-targeted tumors as previously reported by us [40]. Moreover, the treatment of melanoma-bearing mice with Acriflavine, which is reported to prevent the dimerization between HIF-1α and HIF-1β [147], led to an improvement in immunotherapy based on TRP-2 peptide vaccination and anti-PD-1 antibody [40].

Currently, targeting hypoxia, or more particularly HIF-1α, relies on developing hypoxia-activated prodrugs or small inhibitors targeting molecules involved in the survival of hypoxic cells [148].

## 4. Conclusions

Immune checkpoint blockades such as anti-PD-1/PD-L1 and anti-CTLA-4 are promising new strategies that provide durable clinical benefits.

In this review, we briefly overviewed different factors that are involved in the establishment of cold immune desert tumors and new strategies for overcoming immune desert tumors and establishing an inflammatory signature. Converting cold into hot tumors remains the major challenge for improving and extending the clinical benefits of immune checkpoint inhibitors. We believe that such strategies would offer a large variety of therapeutic combination options for improving and extending the benefits of cancer immunotherapy. Among them, the use of neoadjuvant chemotherapy or radiotherapy has provided some encouraging results for improving the infiltration of immune cells within the tumor microenvironment and enhancing the response to immune checkpoint blockade. Targeting autophagy is also an emerging therapeutic option for driving effector T cells to the tumors and improving clinical benefits in combination with immunotherapy in melanoma and colorectal cancer. In addition, oncolytic viruses, nanoparticles, and anti-cancer vaccines are less conventional strategies but nevertheless constitute additional options for switching cold tumors into hot tumors and improving immunotherapy benefits. Finally, metabolic reprogramming by targeting hypoxia is a very promising approach because hypoxia is involved in many steps of cancer biology and impacts the lack of effector immune cells infiltration, the recruitment of immunosuppressive cells, and the up-regulation of inhibitory immune checkpoints. However, HIFs are involved in many pathways and physiological processes—the challenge is to be able to target specifically cancer-related hypoxia without compromising physiological processes that are associated with HIFs. Although several therapeutic options need additional experimental and clinical validation, the strategies described here remain very promising for use in combined immune checkpoint blockades in a large variety of cancers.

## Data Availability

No new data were created or analyzed in this study. Data sharing is not applicable to this article.
